# Supplementary material for: Giant iceberg behaviour impacts regional biogeochemical cycling in the Southern Ocean
Source: Commun Earth Environ. 2026 Apr 20;7(1):353. doi: 10.1038/s43247-026-03440-z (PMC13098799; doi:10.1038/s43247-026-03440-z)
Supplement: Supplementary file 2 — Supplementary material [file 43247_2026_3440_MOESM2_ESM.pdf]

Supplementary information

Giant iceberg behaviour impacts regional biogeochemical cycling in the Southern Ocean

Author list:

Laura T. Taylor[1,2*], Helena Pryer[2], Katharine R. Hendry[1], Rachael N.C. Sanders[1], Michael P. Meredith[1], Andrew Meijers[1], Edward Mawji[3], E. Malcolm S. Woodward[4], Carol Arrowsmith[5], Melanie Leng[5], E Povl Abrahamsen[1], Helen M. Williams[2], Clara Manno[1*]

*Corresponding authors: laulor77@bas.ac.uk, clanno@bas.ac.uk

Affiliations:

1. British Antarctic Survey, High Cross, Madingley Road, Cambridge, CB3 0ET, UK
2. Department of Earth Sciences, University of Cambridge, Downing Street, Cambridge, CB2 3EQ, UK
3. National Oceanography Centre, European Way, Southampton, SO14 3ZH, UK
4. Plymouth Marine Laboratory, Prospect Place, Plymouth, PL1 3DH, UK
5. British Geological Survey, Nicker Hill, Keyworth, NG12 5GG, UK

Supplementary figure S1:

Satellite imagery showing the position and size of iceberg A-23A at four stages of its lifecycle. Panels (a-b) show Landsat images downloaded from USGS EarthExplorer (https://earthexplorer.usgs.gov/), and panels (c-d) show MODIS images downloaded from NASA Worldview (https://wvs.earthdata.nasa.gov/). The measured size of the iceberg in each image is written below, where the blue outline on each image shows the measured shape.

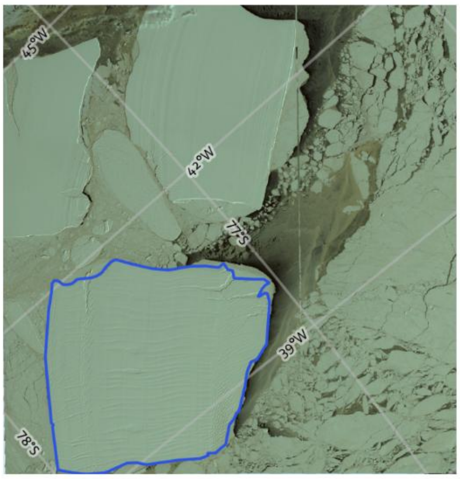

a) 25.10.1987

Size: ~5510 km^2

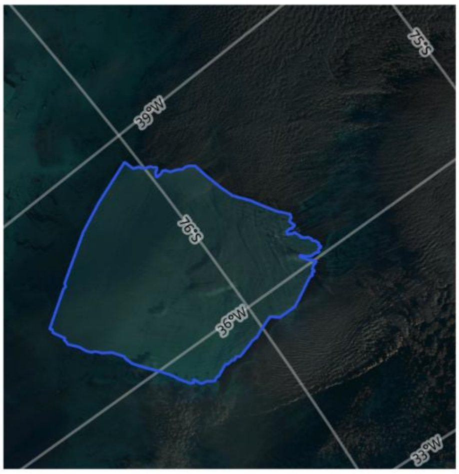

b) 20.03.1989

Size: ~5510 km^2

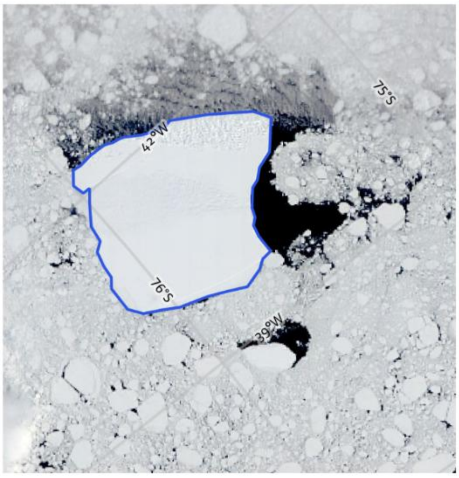

c) 05.01.2020

Size: ~4120 km^2

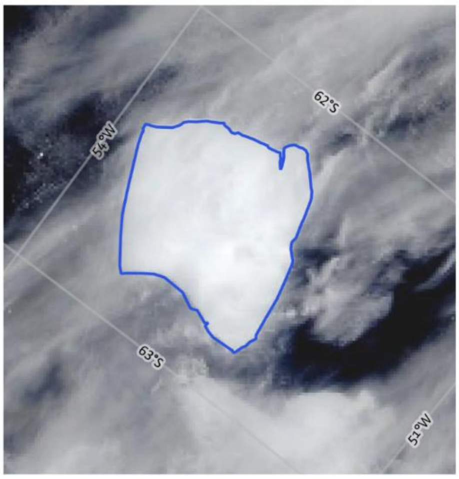

d) 01.12.2023

Size: ~3940 km^2

Supplementary figure S2:

(a) the relationship between salinity and $\delta^{18}O$ (‰) surrounding A-76A (orange) and A-23A (green). The dashed lines represent the gradient between meteoric water and circumpolar deep water endmembers, and the impact of sea ice melt and formation. Circumpolar deep water fraction (%) around A-76A (b) and A-23A (c). $\delta^{18}O$ (‰) around A-76A (d) and A-23A (e).

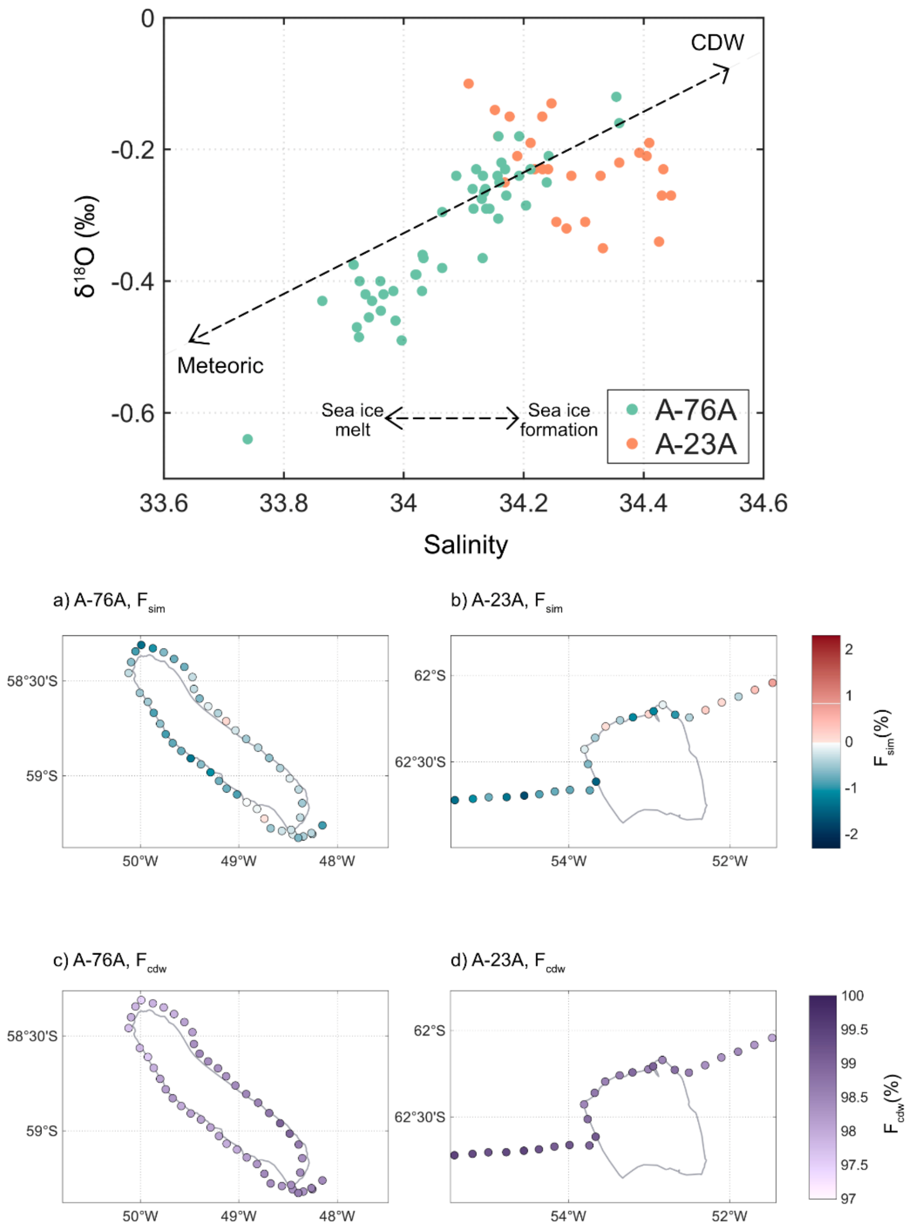

Supplementary note 1

Examination of nutrient stoichiometry with respect to the Redfield ratio

Nutrient supply and utilisation can be assessed using nutrient stoichiometry. The surface ocean nitrate-to-phosphate (N:P) ratio reflects variability in nitrate and phosphate uptake among phytoplankton species [1], while the silicic acid-to-nitrate (Si:N) ratio distinguishes diatom-dominated communities from non-silicifying phytoplankton [2].

These ratios were examined around giant icebergs A-76A and A-23A. The N:P ratio showed a strong positive relationship in both regions, but with a large negative intercept (Figure S1a), potentially reflecting external nutrient inputs or the influence of multiple water masses. In contrast, the Si:N relationship was weak around A-76A and non-significant around A-23A (Figure S1b), with a large positive intercept at A-76A suggesting similar influences. Overall, these stoichiometric ratios had limited utility in constraining nutrient uptake and supply in the vicinity of these icebergs.

Supplementary figure S3:

Relationship between NO_3^- and PO_4^{3-} concentrations (a) and $Si(OH)_4$ and NO_3^- concentrations (b) surrounding icebergs A-76A (green) and A-23A (orange). Linear regressions are fitted for each iceberg, with corresponding equations, coefficients of determination (R^2), and p-values.

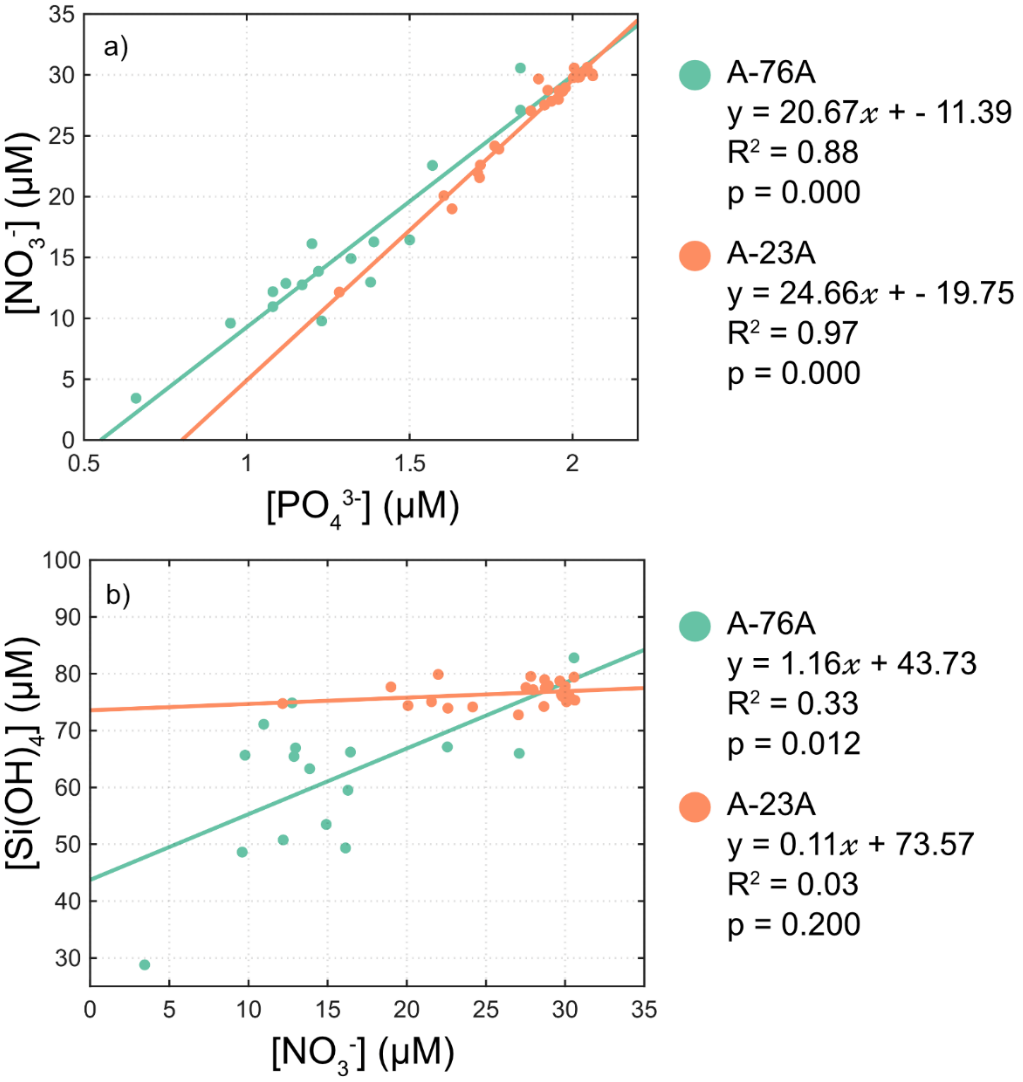

Supplementary table 1:

Correlations between the meteoric water fraction (F_{met}) and biogeochemical variables for icebergs A-76A and A-23A. For each variable, the linear regression equation, coefficient of determination (R^2), and significance (p-value) are shown.

Iceberg	*Variable*	*Equation*	*R^2*	*P-value*
A-76A	NO_3^-	Y = -4.81x + 26.52	0.28	0.042
	NO_2^-	Y = -0.04x + 0.22	0.32	0.029
	PO_4^{3-}	Y = -0.16x + 1.67	0.16	0.141
	DSi	Y = -4.48x + 73.27	0.10	0.253
	N*	Y = -2.32 + -0.19	0.33	0.026
	Si*	Y = 0.33x +46.75	0.00	0.927
	N:P	Y = -2.01x + 16.15	0.38	0.015
	Si:N	Y = 0.99x + 2.20	0.25	0.059
A-76A	NO_3^-	y = 2.75x + 21.80	0.03	0.380
	NO_2^-	y = -0.02x + 0.11	0.04	0.302
	PO_4^{3-}	y = 0.10x + 1.70	0.03	0.414
	DSi	y = 0.98x + 74.86	0.02	0.469
	N*	y = 1.12x + -5.42	0.04	0.357
	Si*	y = -2.00x + 53.32	0.02	0.523
	N:P	y = 0.53x + 13.11	0.01	0.562
	Si:N	y = -0.06x + 3.10	0.00	0.907

Supplementary figure S4:

Closed (top) and open (bottom) system models of $\delta^{30}Si_{DSi}$ fractionation around icebergs A-76A (left) and A-23A (right). Plots display fractionation factors (ε), R^2 values, root mean square error (RMSE) and p-values to illustrate the suitability and fit of each model.

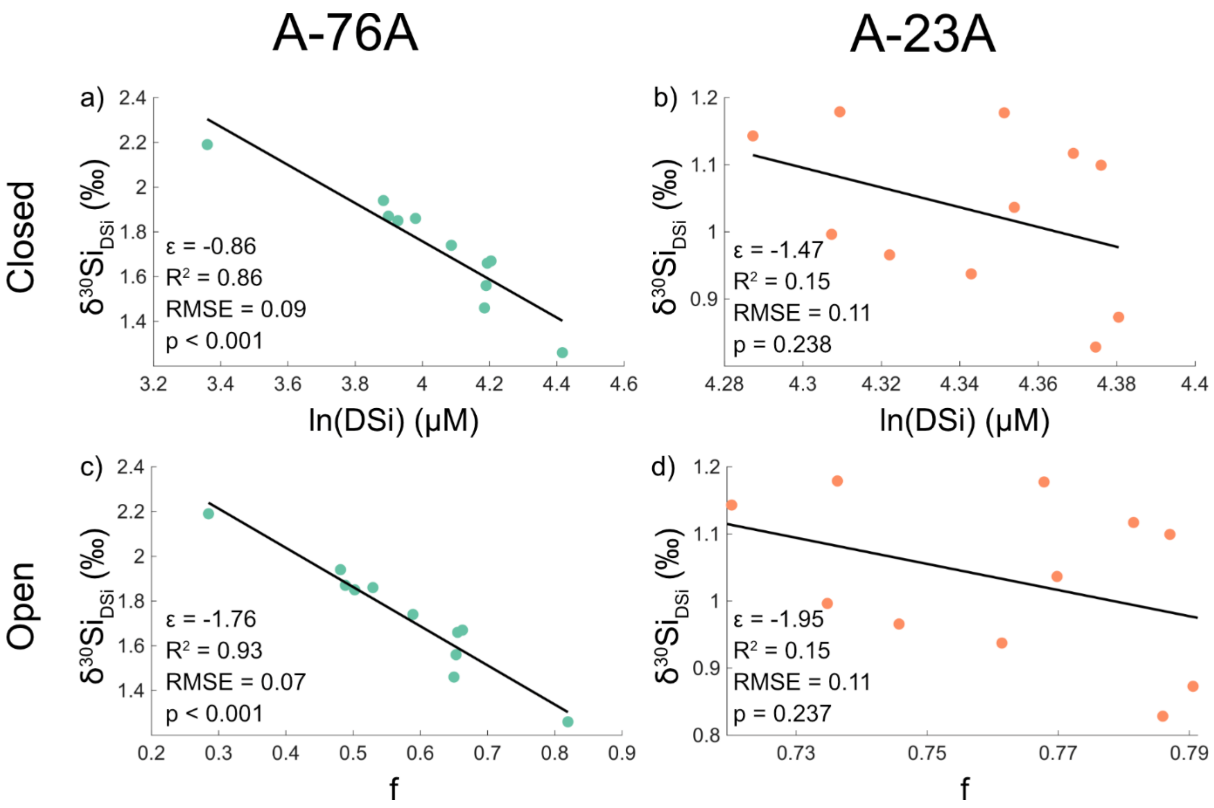

Supplementary table 2:

Salinity and $\delta^{18}O$ (‰) endmembers for sea ice melt, meteoric water, and CDW used in this study, with literature sources.

	Endmember	*Value*	*Citation*
Salinity	Sea ice melt	7	Meredith et al.[3]
	Meteoric water	0	Brown et al. [4]
	Circumpolar deep water	34.73	Meredith et al.[3]
$\delta^{18}O$	Sea ice melt	2.1	Meredith et al. [3]
	Meteoric water	-18	Brown et al. [4]
	Circumpolar deep water	0.1	Meredith et al. [3]

Supplementary references

1. Deutsch, C. & Weber, T. Nutrient Ratios as a Tracer and Driver of Ocean Biogeochemistry. *Annu. Rev. Mar. Sci.* **4**, 113–141 (2012).
2. Sarmiento, J. L., Gruber, N., Brzezinski, M. A. & Dunne, J. P. High-latitude controls of thermocline nutrients and low latitude biological productivity. *Nature* **427**, 56–60 (2004).
3. Meredith, M. P. *et al.* Changing distributions of sea ice melt and meteoric water west of the Antarctic Peninsula. *Deep Sea Res. Part II Top. Stud. Oceanogr.* **139**, 40–57 (2017).
4. Brown, P. J. *et al.* Freshwater fluxes in the Weddell Gyre: results from δ18O. *Philos. Trans. R. Soc. Math. Phys. Eng. Sci.* **372**, 20130298 (2014).
